# Ocean surface waves impact on global air-sea CO${}_2$ flux

**DOI:** 10.1007/s10533-025-01267-y

**Published:** 2025-09-01

**Authors:** Lichuan Wu, Yongqing Cai, Anna Rutgersson

**Affiliations:** 1 Department of Earth Sciences, Uppsala University, Uppsala, 75236 Sweden; 2 Laboratory for Polar Science of the Ministry of Natural Resources, Polar Research Institute of China, Shanghai, 200129 China

**Keywords:** CO$_2$ flux, Bubbles, Wave breaking, Wave-current interactions

## Abstract

Ocean surface gravity waves facilitate gas exchanges primarily in two ways: (1) the formation of bubbles during wave breaking increases the surface area available for gas exchange, promoting CO$_2$ transfer, and (2) wave-current interaction processes alter the sea surface partial pressure of CO$_2$ and gas solubility, consequently affecting the CO$_2$ flux. This study tests these influences using a global ocean-ice-biogeochemistry model under preindustrial conditions. The simulation results indicate that both wave–current interaction processes and the sea-state-dependent gas transfer scheme–which explicitly accounts for bubble-mediated gas transfer velocity–influence the air–sea CO$_2$ flux, with substantial spatial and seasonal variations. In the equatorial region (10$^{\circ }$S–10$^{\circ }$N), both processes enhance the CO$_2$ outgassing flux, with comparable magnitudes (more than 10% on average). However, in the region between approximately 10$^{\circ }$ and 35$^{\circ }$, the impact of ocean surface waves on the air-sea CO$_2$ flux via the sea-state-dependent gas transfer velocity is greater than that of the wave-current interaction processes, with opposing directions of influence. During winter, the sea-state-dependent gas transfer velocity enhances the CO$_2$ uptake flux, while in the summer season, it increases the CO$_2$ outgassing flux. In regions poleward of 35$^{\circ }$, the impact of wave–current interaction processes on CO$_2$ exchange dominates over that of the sea-state-dependent gas transfer velocity. It is worth noting that the impact of wave-current interaction processes on air-sea CO$_2$ flux is primarily driven by changes in the ratio between the concentrations of dissolved inorganic carbon and total alkalinity, with variations in sea surface temperature exerting an opposite influence on pCO$_2$, albeit with a smaller magnitude. Overall, wave-related processes should be considered in Earth System Models to better model the carbon cycle.

**Supplementary Information:**

The online version contains supplementary material available at 10.1007/s10533-025-01267-y.

Introduction

The exchange of CO$_2$ between the atmosphere and the ocean plays a crucial role in the global carbon cycle and climate change. Approximately one-quarter of the CO$_2$ emissions in recent years have been absorbed by the ocean (Friedlingstein et al. 2022; Holzer and DeVries 2022). However, the ocean’s uptake or release of carbon to the atmosphere varies with seasons and regions (Watson and Orr 2003). Therefore, accurately representing the air-sea CO$_2$ flux is essential for describing ocean biogeochemical processes, global carbon cycle, and climate projection, as CO$_2$ is the major anthropogenic greenhouse gas (e.g., Wanninkhof 2014).

The air-sea CO$_2$ flux is controlled by the air-sea difference in partial pressure, gas solubility, and the efficiency of the exchange, which is often described by:

1 $$\begin{aligned} F = K_0 k_w \Delta p_{co_2} \end{aligned}$$

where $K_0$ is the gas solubility which varies with sea surface temperature (SST) and surface salinity (SSS) (Weiss 1974). $\Delta p_{co_2}$ is the air-sea CO$_2$ partial pressure difference and mainly responds to changes in the ocean biogeochemical processes and the concentration of CO$_2$ in the atmosphere. The gas transfer velocity, $k_w$, represents the efficiency of CO$_2$ transfer at the air-sea interface and is mainly controlled by processes disturbing the ocean-side molecular sublayer (e.g., Garbe et al. 2014). In this study, a positive value of *F* represents the carbon outgassing, and vice versa. A range of processes potentially influences the ocean-side molecular sublayer including convection, surfactants, bubbles and sea spray (Rutgersson et al. 2011; Woolf et al. 2019; Ribas-Ribas et al. 2018; Andreas et al. 2016; Gutiérrez-Loza et al. 2022), but for the global oceans it is particularly associated with upper ocean turbulence (e.g., Esters et al. 2017; Gutiérrez-Loza et al. 2022). Surface wind speed, as a proxy for the intensity of upper ocean turbulence, is widely used to parameterize CO$_2$ transfer velocity (Liss and Slater 1974; Wanninkhof 2014). This type of parameterization is commonly employed in state-of-the-art climate models and in estimating the global air-sea CO$_2$ flux (e.g., Takahashi et al. 2009). However, wind speed-dependent parameterizations of $k_w$ vary across different datasets (Wanninkhof 2014; Rustogi et al. 2024; McGillis et al. 2001). This suggests that other factors besides wind speed may play important roles in determining air-sea CO$_2$ transfer velocity. Consequently, using a wind speed-dependent parameterization may introduce potential biases into climate models.

Breaking waves are known to entrain air bubbles which can substantially increase the surface area available for gas exchange, facilitating the gas exchange at the air-sea interface (Bell et al. 2017; Liang et al. 2013). Under moderate to high wind speeds, the presence of air bubbles is most prominent during wave breaking at the ocean surface (Deike 2022). Consequently, wind speed-dependent parameterizations exhibit significant scatter due to bubble-mediated gas transfer (Edson et al. 2011; Garbe et al. 2014). To address this issue, some studies have attempted to separate total gas transfer into bubble-mediated and non-breaking wave-induced components (Deike and Melville 2018) where the bubble-mediated transfer velocity is directly determined by wave-breaking information. This approach reduces the uncertainty of air-sea gas transfer velocity, particularly during moderate to high winds (Deike and Melville 2018). With the parameterization, it is estimated that bubble-mediated processes account for approximately 40% of the net air-sea flux, with significant regional and seasonal variation (Reichl and Deike 2020). Compared to the wind speed-dependent parameterization, this expanded approach can significantly increase the air-sea CO$_2$ flux, enhancing ocean CO$_2$ uptake in regions such as the Southern Indian Ocean (Sun et al. 2023).

In addition to influencing gas transfer velocity by the entrainment of air bubbles, ocean surface gravity waves can also indirectly impact the air-sea CO$_2$ flux by altering sea surface pCO$_2$, gas solubility, and gas transfer velocity via changes in Schmidt number (Eq. 2). These effects are primarily due to changes in SST, SSS, dissolved inorganic carbon (DIC), and total alkalinity (ALK) caused by wave-current interaction processes. These processes include the impacts of waves on wind stress, Langmuir circulation, Coriolis-Stokes forcing, and nonbreaking wave-induced mixing (Wu et al. 2015, 2016, 2024; Hasselmann 1970; Li et al. 2019; Reichl et al. 2016; Zhou et al. 2023). Incorporating these wave-related processes significantly improves the climate model’s ability to capture mixed layer depth, ventilation, SST, and more (Law Chune and Aouf 2018; Li et al. 2016; Fan and Griffies 2014; Qiao et al. 2004). These processes not only influence physical parameters but also affect biogeochemical parameters, i.e., the carbon cycle, as they are tightly coupled. However, over the past decade, the indirect influence of ocean surface waves on biogeochemical processes has been less explored compared to their direct impact on physical processes.

In this study, we investigate the effects of wave-driven parameterizations of (i) gas transfer velocity and (ii) upper-ocean mixing and transport on the air-sea CO$_2$ flux. Different from previous studies (e.g. Sun et al. 2023) that estimate the air-sea gas transfer velocity or CO$_2$ flux offline, we test the wave influence on air-sea CO$_2$ flux in a coupled ocean-ice-biogeochemistry model to better capture the feedback effects. In the experiment incorporating sea-state-dependent gas transfer velocity in this study, the influence of bubbles on the CO$_2$ transfer velocity is explicitly accounted for in the parameterization (Deike and Melville 2018). To assess the influence of upper-ocean mixing and transport on air-sea CO$_2$ fluxes–through modifications to sea surface pCO$_2$, gas solubility, and gas transfer velocity–various wave–current interaction schemes are implemented into the model. To achieve the study’s aim, we tested these influences under preindustrial conditions, based on sensitivity experiments. The remainder of the paper is organized as follows: Section 2 introduces the parameterization employed in this study, Section 3 describes the model setup and experiments, Section 4 presents the simulation results, and Sections 5 and 6 provide the discussion and conclusions, respectively.

## Parameterizations

### Wind speed dependent gas transfer velocity

The air-sea gas transfer velocity is commonly estimated based on expressions using a squared wind speed dependence (e.g., Wanninkhof 2014; McGillis et al. 2001). Here we use the following

2 $$\begin{aligned} k_w = T \times U_{10}^2 \left( \frac{Sc}{660} \right) ^{-1/2} \end{aligned}$$

where *T* is the constant varying with studies, in this study $T=0.251 (cm~hr^{-1})(m s^{-1})^{-2}$ (Wanninkhof 2014), $U_{10}$ is the mean wind speed at 10 m above the sea surface, and *Sc* is the Schmidt number. This wind-speed-dependent parameterization is employed in the reference simulation to evaluate the impact of wave-driven parameterizations on air-sea CO$_2$ fluxes, through comparison with the results of other sensitivity experiments.

### Sea-state-dependent gas transfer velocity

Under high wind, the formation of bubbles during wave breaking increases the surface area available for gas exchange, further promoting CO$_2$ transfer. Thus, the air-sea CO$_2$ gas transfer velocity can be divided into two components, 1) the nonbreaking gas transfer due to molecular diffusion linked to the ocean turbulence, and 2) the bubble-mediated gas transfer component associated with wave breaking. The total air-sea CO$_2$ gas transfer velocity can be estimated by the sum of the two components (Deike and Melville 2018; Zhou et al. 2023)

3 $$\begin{aligned} k_w = k_{nb} + k_{b} = A_{NB} u_* \left( \frac{Sc}{660} \right) ^{-1/2} + \frac{A_B}{\alpha ^{0.35}} \left[ u_*^{5/3} \sqrt{gH_s}^{4/3} \right] \left( \frac{Sc}{660} \right) ^{-1/2} \end{aligned}$$

where $A_{NB}=1.55 \times 10^{-4}$ is a nondimensional coefficient obtained from measurements, $u_*$ is the air friction velocity estimated by COARE algorithm (Fairall et al. 2003), $A_B=1.0 \pm 0.2 \times 10^{-5} s^2m^{-2}$ is a fitting coefficient, $\alpha$ is Ostwald dimensionless solubility coefficient, *g* is the acceleration of gravity, and $H_s$ is the significant wave height.

### Wave-current interaction processes

#### Sea-state dependent water-side stress

Traditionally, the water-side stress, i.e., the momentum flux to the ocean column, is assumed to be identical to the air-side stress, i.e., the momentum flux lost from the atmosphere. However, due to the influence of ocean surface waves, the air-side stress can differ from the water-side stress. At the air-sea interface, a fraction of the momentum flux lost from the atmosphere forces the development of ocean surface waves. The rest of the momentum flux will directly go to the ocean currents. When waves break, they transfer momentum flux from waves to the ocean column. Considering this buffer role of ocean surface waves in the air-sea momentum flux, the momentum conservation can be described by (ECMWF 2020; Wu et al. 2022)

4 $$\begin{aligned} \varvec{\tau }_\textrm{oc} = \varvec{\tau }_\textrm{a} - \varvec{\tau }_\textrm{w} - \varvec{\tau }_\textrm{ds}, \end{aligned}$$

where, $\varvec{\tau }_\textrm{oc}$ is the momentum flux to the ocean column, $\varvec{\tau }_\textrm{a}$ is the momentum flux lost from the atmosphere, $\varvec{\tau }_\textrm{w}$ is the momentum flux from the atmosphere to generate ocean surface waves, and $\varvec{\tau }_\textrm{ds}$ (negative term) is the momentum flux from surface waves to the ocean currents through breaking. $\varvec{\tau }_\textrm{a} - \varvec{\tau }_\textrm{w}$ is the momentum flux across the air-sea interface which is not used to generate waves. Both $\varvec{\tau }_\textrm{w}$ and $\varvec{\tau }_\textrm{ds}$ are determined by 2D wave spectrum. Therefore,

5 $$\begin{aligned} \varvec{\tau }_\textrm{oc} = \varvec{\tau }_\textrm{a} - \rho _\textrm{w} g \int _{0}^{2\pi } \! \int _{0}^{\infty } \frac{\textbf{k}}{\omega } (S_\textrm{in} + S_\textrm{ds}) \, d\omega d\theta , \end{aligned}$$

where $S_\textrm{ds}$ and $S_\textrm{in}$ represent the dissipation and the wind input source terms in a wave model respectively, $\omega$ is the wave angular frequency, and $\theta$ is the wave direction.

Neglecting the direction difference between $\varvec{\tau }_\textrm{a}$ and $\varvec{\tau }_\textrm{oc}$, the water-side wind stress can be estimated by (Janssen 2012; Wu et al. 2024)

6 $$\begin{aligned} \varvec{\tau }_\textrm{oc} = \beta \varvec{\tau }_\textrm{a} \end{aligned}$$

where $\beta = \frac{|\tau _\textrm{oc,w}|}{|\tau _\textrm{a,w}|}$ is the normalized stress calculated from a wave model based on the momentum conservation equation (Eq. 5). $\tau _\textrm{oc,w}$ and $\tau _\textrm{a,w}$ are the water-side and air-side stress calculated in a wave model. Under growing waves, the momentum flux from the atmosphere to waves, $\varvec{\tau }_\textrm{w}$, is larger than the momentum flux from waves to the oceanic interior though wave breaking, $\varvec{\tau }_\textrm{ds}$, leading to $\beta <1$. In contrast, for decaying waves, $\varvec{\tau }_\textrm{ds}$ is larger than $\varvec{\tau }_\textrm{w}$, resulting $\beta >1$.

#### Stokes drift related processes

Stokes drift due to ocean surface waves can affect the upper ocean simulation through the Coriolis-Stokes force (CSF) and vortex force, as well as Stokes advection. By taking these Stokes drift related processes, the governing equations of Eulerian ocean models become

7 $$\begin{aligned} &  \frac{D\textbf{u}}{Dt} = -\frac{1}{\rho _\textrm{w}}\nabla p - \underbrace{(\textbf{u}_\textrm{s} \cdot \nabla ) \textbf{u}}_\mathrm {Stokes \ adv} + \textbf{u} \times f \hat{\textbf{z}} + \underbrace{\mathbf {u_\textrm{s}} \times f \hat{\textbf{z}}}_\textrm{CSF} + \underbrace{ \textbf{u}_\textrm{s} \times \zeta }_\mathrm {Vortex \ force} + \mathbf {{D}}^\textrm{u} - g\hat{\textbf{z}} \end{aligned}$$

8 $$\begin{aligned} &  \frac{Dc}{Dt} = -\underbrace{ \mathbf {{u}}_\textrm{s} \cdot \nabla c }_\mathrm {Stokes \ adv} + \mathbf {{D}}^\textrm{c} \end{aligned}$$

9 $$\begin{aligned} &  \frac{\partial p}{\partial z} = -\rho _\textrm{w} g \end{aligned}$$

10 $$\begin{aligned} &  \nabla \cdot \textbf{u} = 0 \end{aligned}$$

11 $$\begin{aligned} &  \nabla \cdot \textbf{u}_\textrm{s} = 0 \end{aligned}$$

12 $$\begin{aligned} &  \frac{\partial \eta }{\partial t} = -\nabla _\textrm{h} \int _{z=-H}^{z=\eta } (\textbf{u}_\textrm{h} + \textbf{u}_\textrm{s}) \, dz. \end{aligned}$$

Here, $\textrm{u}$ is the ocean current, *t* is the time, $\rho _w$ is the water density, *p* is the water pressure, $\mathbf {{u}}_\textrm{s}$ is the Stokes drift, *f* is the Coriolis parameter, $\zeta$ is the mean flow vorticity, $\mathbf {{D}}^\textrm{u}$ and $\mathbf {{D}}^\textrm{c}$ represent the sub-grid scale physical processes for momentum and tracer equations, *c* is a scalar quantity, $\eta$ is the sea-surface height, $\textrm{h}_h$ is the horizontal current vector, *g* is the gravitational acceleration with $\hat{\textbf{z}}$ the vertical unit vector, $\frac{D}{Dt} = \frac{\partial \textbf{u} }{\partial t} + \textbf{u} \cdot \ \nabla \textbf{u}$ represent the material derivative. For details of these processes in the NEMO model, readers refer to Couvelard et al. (2020) and Wu et al. (2019).

In the *k*–$\epsilon$ turbulence closure scheme, the vertical diffusion used for calculating the subgrid momentum flux is estimated based on the turbulence kinetic energy (TKE) equation. To account for the enhancement of upper-ocean mixing by Langmuir circulation, an additional source term in the turbulence kinetic energy (TKE) equation is added, following (Axell 2002),

13 $$\begin{aligned} P_{LC} (z) = \frac{w^3_{LC}(z)}{H_{LC}} \end{aligned}$$

where $H_{LC}$ is the depth of Langmuir circulation and $w_{LC}(z)$ is the vertical velocity profile of Langmuir circulation,

14 $$\begin{aligned} w_{LC} = c_{LC} u_{s0}^{LC} sin \left( -\frac{\pi z}{H_{LC}} \right) , 0<z<H_{LC}. \end{aligned}$$

For details on the turbulence closure scheme and the calculation of $H_{LC}$, see Axell (2002). Different from the original scheme developed by Axell (2002), the influence of the misalignment between Stokes drift and the wind direction on the Langmuir cells is considered in this study (Couvelard et al. 2020),

15 $$\begin{aligned} u_{s0}^{LC} = max(u_{s0} \cdot e_\tau , 0) \end{aligned}$$

where $u_{s0}$ is the surface Stokes drift and $e_\tau$ is the unit vector in the wind stress direction. The coefficient $c_{LC}$ is set to 0.15 in this study. This scheme has been tested in global ocean simulations in previous studies (e.g., Breivik et al. 2015)

## Models setup and experiments

### NEMO model setup

The “Nucleus for European Modelling of the Ocean” (NEMO) model version 4.2.2 (Madec et al. 2017) is utilized in this study for sensitivity simulations, which includes an ocean module, a sea ice module, and a passive tracer module and a biogeochemical module, PISCES (Pelagic Interaction Scheme for Carbon and Ecosystem Studies). To save computational resource, the ORCA2 global configuration is used in this study, which is a tripolar grid with a horizontal resolution of 2$^{\circ }$x2$^{\circ }$ cos(latitude). In total, there are 31 vertical levels with 15 vertical layers in the upper 200m. The ORCA2_ICE_PISCES configuration in the NEMO code serves as the reference setup. The TKE turbulent closure scheme is used in the ocean model (Blanke and Delecluse 1993). The COARE 3.6 algorithm (Edson et al. 2013) is used to estimate the air-sea turbulence flux since the nonbreaking gas transfer velocity ($k_{nb}$ in Eq. 3) in Deike and Melville (2018) is estimated based on the COARE algorithm. All the other setups in the control experiment are the same as the ORCA2_ICE_PISCES configuration.

### Wave model setup

The WaveWatch-III (WW3; (Tolman 2009)) model is used for wave simulation providing the wave information (i.e., $H_s$, $\beta$, and $\mathbf {u_{s0}}$) needed by the model. The WW3 domain covers latitudes between -80$^{\circ }$ and 80$^{\circ }$ with a horizontal resolution of 1 degree. The ST4 source term package (Ardhuin et al. 2010) is employed in the simulation, with wave-ice interaction parameterization switched off. The frequency spectrum is discretized into 29 frequencies, starting at 0.035 Hz with an increase factor of 1.1. The directional resolution is 15 degrees. ETOPO data (Amante and Eakins 2009) are used for topography. The wind information from the Common Ocean-ice Reference Experiments v2 (CORE v2) dataset (Large and Yeager 2009) and sea ice information from the mean of the NEMO control simulation are used to drive the wave model. Since the normal year of forcing is used, one year wave simulation data after one-month spun up are used to provide the necessary wave information for the NEMO simulations.

**Fig. 1:**
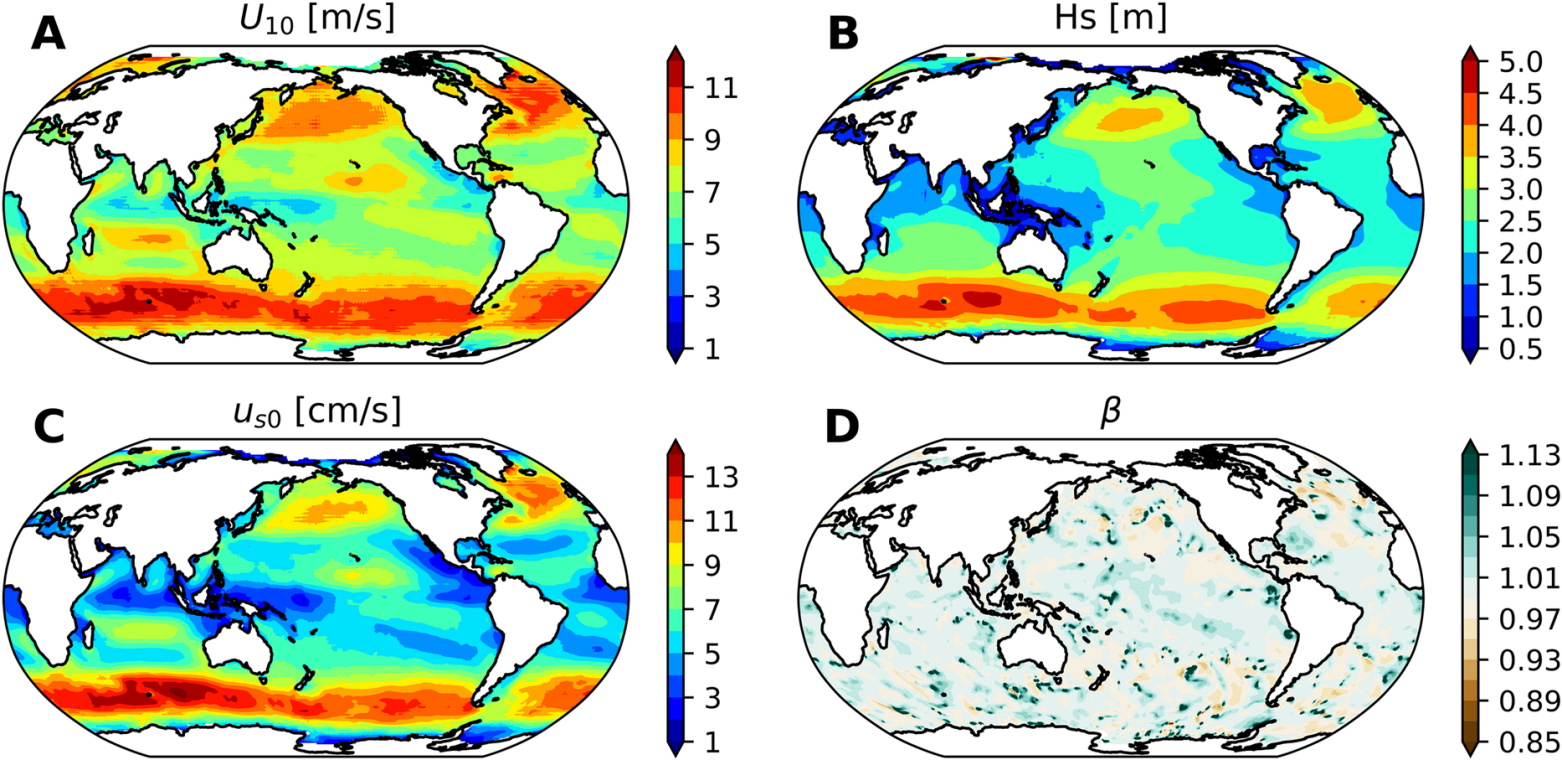
The distribution of mean wind speed [unit: m/s] (A), (B) mean significant wave height [unit: m], (C) mean surface Stokes drift [unit: cm/s], and (D) snapshot of the normalized stress $\beta$ at 25th January 00:00

The mean wind speed, significant wave height, surface Stokes drift, and a snapshot of the normalized stress are shown in Fig. 1. The mean wind speed exceeds 10 m/s in the Southern Ocean (Fig. 1A), with a surface significant wave height of more than 4 m (Fig. 1B) and a surface Stokes drift higher than 11 cm/s (Fig. 1C). In the Northern Hemisphere, areas with high wind and wave activity are primarily located in the mid- and high-latitude oceans, corresponding to storm regions. Wave height and surface Stokes drift are low in tropical areas, with the lowest values found in the central Pacific Ocean and the northern Indian Ocean. A snapshot of the normalized stress, $\beta$, is shown in Fig. 1D, which varies with sea state (growing or decaying). For detailed climatological information on $\beta$, refer to Wu et al. (2022).

### Experiments

To explore the influence of different wave-related approaches on the air-sea CO$_2$ flux, four experiments are designed (Table 1):

- **CTL experiment**: In the control experiment, the wind speed-dependent gas transfer velocity parametrization (Eq. 2) is used. All the wave-current interaction processes listed in Section 2.3 are switched off.
- **WaveCO2 experiment**: In this experiment, the sea-state-dependent gas transfer velocity, i.e., the parameterization explicitly accounts for the bubble-mediated transfer velocity (Eq. 3), is used to estimate the air-sea CO$_2$ flux in this experiment. All other configurations of WaveCO2 are identical to those in the CTL experiment.
- **WaveMix experiment**: In this experiment, the wave-current interaction processes listed in Section 2.3 are switched on, i.e., wave impact on wind stress, CSF and vortex force, Stokes advection, and Langmuir circulation (Eq. 7-12 and Eq. 13). The wave simulation provides the wave parameters needed by the wave-current interaction processes. The air-sea CO$_2$ transfer velocity is calculated using the wind speed dependent only parametrization (Eq. 2).
- **FULL experiment**: In this experiment, both wave–current interaction processes (Eq. 7-12 and Eq. 13) and the sea-state-dependent air–sea gas transfer velocity (Eq. 3)–explicitly accounting for bubble-mediated transfer–are implemented into the model.

With the four experiments, we can identify: 1) the influence of sea-state-dependent gas transfer velocity–explicitly accounting for bubble-mediated transfer–on air-sea CO$_2$ flux by comparing WaveCO2 and CTL, 2) the influence of wave-current interactions on air-sea CO$_2$ flux by comparing WaveMix and CTL, and 3) the combined influence of wave-dependent parameterizations–namely, sea-state-dependent gas transfer velocity and wave–current interaction processes–on the air–sea CO$_2$ flux.

**Table 1:** The designs of the experiments

Experiments	CTL	WaveCO2	WaveMix	FULL
Wind-speed dependent only gas transfer velocity , Eq. 2 (Wanninkhof 2014)	Yes		Yes	
Explicitly include the bubble-induced CO2 transfer velocity, Eq. 3 (Deike and Melville 2018)		Yes		Yes
Include the impact of wave-current interactions: 1) Sea-state dependent water-side stress, Eq. 4 (Janssen 2012); 2) Stokes drift related processes, Eq. 7-12, (Couvelard et al. 2020) and Langmuir circulation, Eq. 13 (Axell 2002)	No	No	Yes	Yes

The initial temperature and salinity for the NEMO model are derived from the World Ocean Atlas 2009 climatology. The data used to initialize the biogeochemical model PISCES include ALK, DIC, dissolved organic concentration, dissolved iron concentration, nitrates concentration, dissolved oxygen concentration, phosphate concentration, and silicate concentration. These data are obtained from the SETTE (System Evaluation Test for NEMO) framework (https://gws-access.jasmin.ac.uk/public/nemo/sette_inputs/). The CORE v2 normal year forcing is employed as the forcing data. The CORE dataset is widely used for mean climate simulations of ocean and sea ice (Large and Yeager 2009). The model runs for 300 years for the CTL experiment. Given that our study focuses on upper ocean circulation, a 300-year simulation period is deemed sufficient (Yeager et al. 2012; Danabasoglu et al. 2014). In the simulations, the atmospheric pCO$_2$ is kept constant at 280 ppm everywhere in the globe, which is the preindustrial value. For the WaveCO2, WaveMix, and FULL experiments, the model is integrated for 60 years, restarting from the CTL experiment at the end of the 240 years. All the following analyses are based on the 60-year mean unless otherwise specified. It should be noted that simulations with a 60-year deviation from the control run (CTL) may be subject to model drift. However, since the aim of this study is to explore the role of waves in the air-sea CO$_2$ flux, this configuration is expected to have minimal impact on the results.

**Fig. 2:**
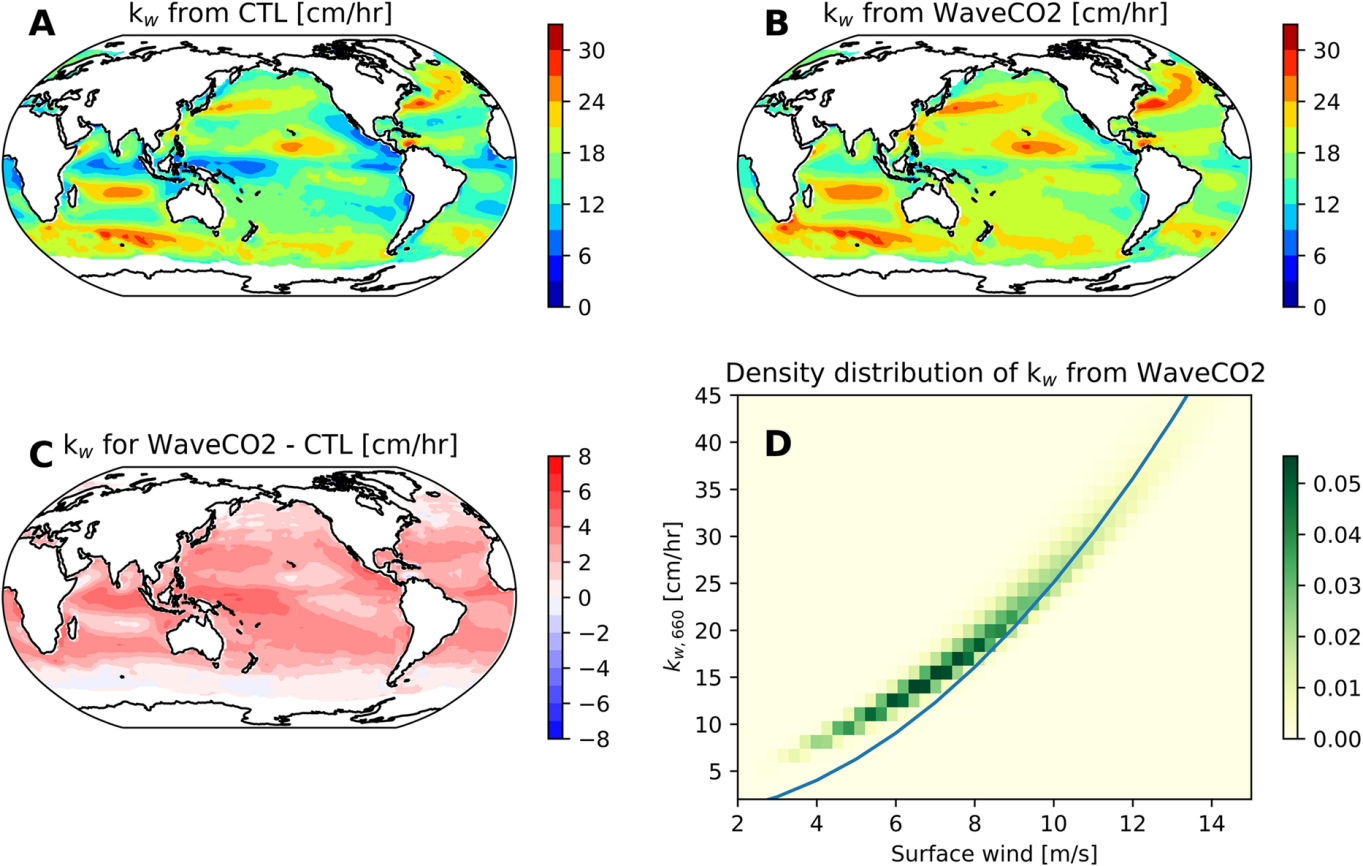
Distribution of the mean gas transfer velocity from (A) CTL and (B) WaveCO2, and (C) the difference between WaveCO2 and CTL. Panel (D) shows the normalized density distribution of the gas transfer velocity across different wind speed bins from WaveCO2. The solid blue line in (D) represents the results from CTL (i.e., Eq. 2)

## Results

### CO$_2$ transfer velocity

The mean CO$_2$ transfer velocity, $k_w$, from CTL and WaveCO2, is shown in Figs. 2A and B, respectively. The $k_w$ parameterization used in WaveMix is the same as in CTL, whereas the $k_w$ applied in FULL is identical to the one used in WaveCO2. Therefore, their differences are minimal and primarily due to variations in the Schmidt number, which are caused by SST variations resulting from wave-current interactions. It is evident that $k_w$ varies with location, exhibiting higher values in areas with high wind speeds (Fig. 1A). Relative to CTL, the mean $k_w$ from WaveCO2 is generally higher across most regions (Fig. 2C), with the most pronounced differences occurring near the equator. The density distribution of the normalized transfer velocity, $k_{w,660}=k_w (\frac{660}{sc})^{-1/2}$, as a function of surface wind speed (Fig. 2D) shows that $k_{w,660}$ from WaveCO2 is consistently higher than that from CTL when wind speeds below 10 m/s (the solid blue line represents the results from CTL). The large scatter in $k_{w,660}$ values observed in WaveCO2 highlights the influence of sea state variability.

The influence of sea state impact on the CO$_2$ gas transfer velocity and its global spatial distribution has been widely explored in previous studies. Readers are referred to Zhou et al. (2023), which found that the gas transfer velocity parameterization explicitly considering the bubble contribution generally agrees better with measurements and reduces the underestimation of gas transfer velocity compared to traditional wind-speed–only–based parameterizations. Additionally, the enhanced gas transfer velocity due to bubbles is frequently observed in areas with developed sea states. In this study, we will not repeat the same analysis. Instead, we focus on the CO$_2$ flux and the parameters affecting pCO$_2$ in the following subsections.

### CO$_2$ flux and pCO$_2$

**Fig. 3:**
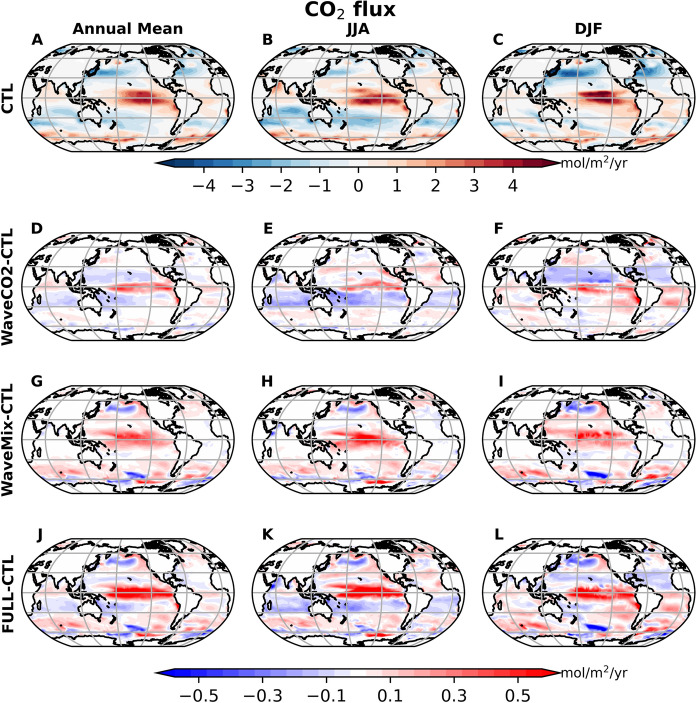
The mean CO$_2$ flux from the CTL experiment (the first row). The second, third, and fourth rows are the differences between the sensitivity experiment and CTL experiment, WaveCO2-CTL, WaveMix-CTL, and FULL-CTL, respectively. The three columns represent the annual mean, JJA (June-July-August) mean, and DJF (December-January-February) mean, respectively [unit: mol/m$^2$/yr]. In panels A–C, blue (red) indicates carbon uptake (outgassing). In the other panels, red shading denotes experimental runs with either increased CO$_2$ outgassing flux or reduced carbon uptake flux, and vice versa for blue

**Fig. 4:**
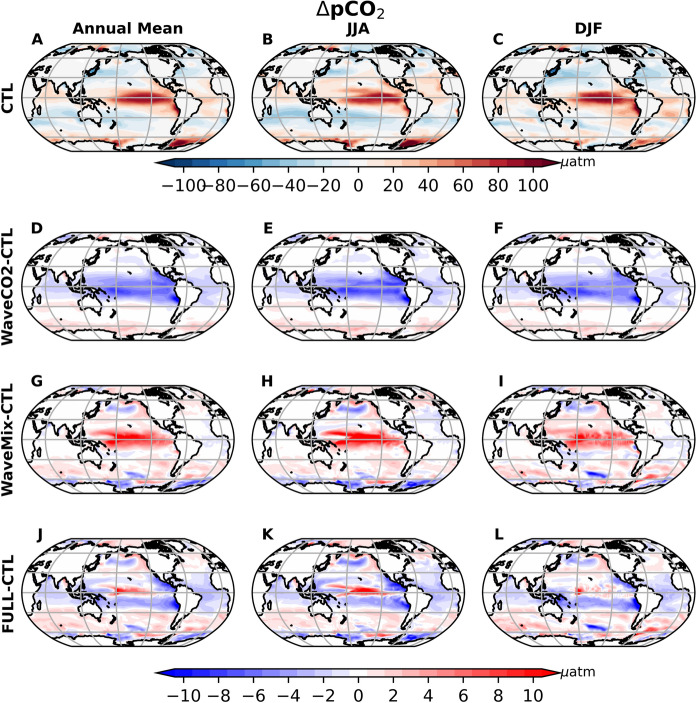
The $\Delta$pCO$_2$ from the control experiment (the first row) and the difference between the sensitivity experiment with CTL: the second, third, and fourth rows represent WaveCO2-CTL, WaveMix-CTL, and FULL-CTL, respectively. The first to third columns represent the annual mean, JJA mean, and DJF mean. In panels D–L, blue indicates experimental runs with smaller ocean surface pCO$_2$ than CTL, corresponding to reduced $\Delta$pCO$_2$, while red indicates the opposite. unit: $\mu$atm

The CO$_2$ flux exhibits both spatial and temporal variability, as shown in Fig. 3. The carbon outgassing is predominantly found in the equatorial region (warm color in Fig. 3A) and carbon uptake is prevalent in mid and high latitudes (cold color in Fig. 3A). The high carbon outgassing flux around 20-50 degrees has significant seasonal variation, with the largest carbon outgassing flux value in the winter and early spring season, i.e., December-January-February-March in the Northern Hemisphere and June-July-August-September-October in the Southern Hemisphere (Figs. 3B-C, and Fig. S1A in the supporting information). This seasonal variation is mainly resulted from the seasonal variation of the wind speed (the figure is not shown) and sea surface CO$_2$ partial pressure (Figs. 4B and C), pCO$_2$.

Compared to the control experiment (CTL), WaveCO2 generally increases carbon outgassing in equatorial regions (approximately 10$^\circ$S-10$^\circ$N) throughout the year (the area with red color in Figs. 3D-F and S1B). This occurs despite a reduction in $\Delta$pCO$_2$ in WaveCO2 (blue areas in Fig. 4D–F), which would otherwise act to suppress the carbon outgassing flux. This indicates that the enhanced $k_w$ is the dominant factor driving the increase in carbon outgassing in this region. Surrounding this band, WaveCO2 increases the carbon update (or decrease the carbon outgassing) (blue color area in Fig. 3D-F) in areas extending to 35$^\circ$N and 35$^\circ$S primarily during the winter and early spring seasons (Figs. 3E-F, and S1). Both the reduction in $\Delta$pCO$_2$ (Fig. 4D–F) and the enhancement of $k_w$ (Fig. 2A–B) contribute to the increased carbon uptake flux. Conversely, during July, August, and September in the Northern Hemisphere (corresponding to December, January, and February in the Southern Hemisphere), $\Delta$pCO$_2$ is reduced in the WaveCO2 experiment, indicating a decrease in the partial pressure of CO$_2$ in the ocean. This shift enhances the carbon uptake. At the same time, the elevated gas transfer velocity further supports this enhancement of the carbon uptake. In the Southern Ocean, WaveCO2 generally reduces carbon outgassing flux at latitudes higher than about 60$^\circ$S throughout the year. This reduction is primarily driven by the change of $k_w$, as the increase in surface ocean partial pressure of CO$_2$ enhances the CO$_2$ outgassing flux (Fig. 4D–F). In the Arctic, WaveCO2 generally reduces the CO$_2$ uptake flux (Fig. S2), primarily due to differences in $k_w$ between the WaveCO2 and CTL experiments.

Adding wave-current interaction processes (WaveMix) generally enhances the carbon outgassing flux or decreases the carbon uptake flux (area with red color in Figs. 3G-I and positive values in Fig. S1). However, south of 50$^{\circ }$S, WaveMix reduces the carbon outgassing from January to June but enhances it from September to December. These changes are caused by variations in the sea surface pCO$_2$ (Fig. 4G-I), which are directly linked to the variation of DIC, ALK, SST, and sea ice extent. These factors are directly affected by wave-current interaction processes, which will be discussed separately in the following subsections.

When both wave-related processes are included in the model (FULL), the CO$_2$ outgassing flux in the equatorial region increases by more than 10% compared to the control run (CTL), exceeding the enhancement from including only one process (Figs. 3J-L and S1B). In the winter hemisphere, between approximately 10$^\circ$ and 35$^\circ$, the FULL experiment enhances the CO$_2$ uptake flux. The enhancement results from opposing effects: wave impacts on gas transfer enhance the CO$_2$ uptake flux, while wave-current interaction processes tend to suppress it. The net difference between FULL and CTL follows the same direction as that between WaveCO2 and CTL, but with a smaller magnitude (Figs. 3 and S1).

### DIC and ALK

DIC and ALK play a crucial role in regulating pCO$_2$ at the sea surface. An increase in DIC enhances the concentration of dissolved CO$_2$ in seawater, thereby raising pCO$_2$. In contrast, higher ALK acts as a buffer in the carbonate system, shifting the chemical equilibrium toward bicarbonate (HCO$_3^-$) and carbonate (CO$_3^{2-}$) ions. This reduces the concentration of dissolved CO$_2$ gas, leading to lower pCO$_2$ levels in surface waters. In NEMO-PISCES, DIC and ALK are treated as tracer variables that are advected and diffused by the ocean circulation model. Consequently, physical processes such as wave–current interactions can influence their distribution by modifying vertical mixing and advection. Additionally, biogeochemical source and sink terms also affect DIC and ALK. For example, during CO$_2$ outgassing, the system shifts to replenish CO$_2$ by converting HCO$_3^-$ and CO$_3^{2-}$, thereby reducing DIC. Conversely, during CO$_2$ uptake, atmospheric CO$_2$ dissolves in seawater and reacts with water to form carbonic acid, which dissociates into HCO$_3^-$ and CO$_3^{2-}$, increasing DIC. Surface ocean pCO$_2$, by extension, to the air–sea CO$_2$ flux, is sensitivity to the ratio of DIC to ALK (Mongwe et al. 2016). In the rest of this subsection, the impact of DIC/ALK and its response to wave-related processes will be examined.

**Fig. 5:**
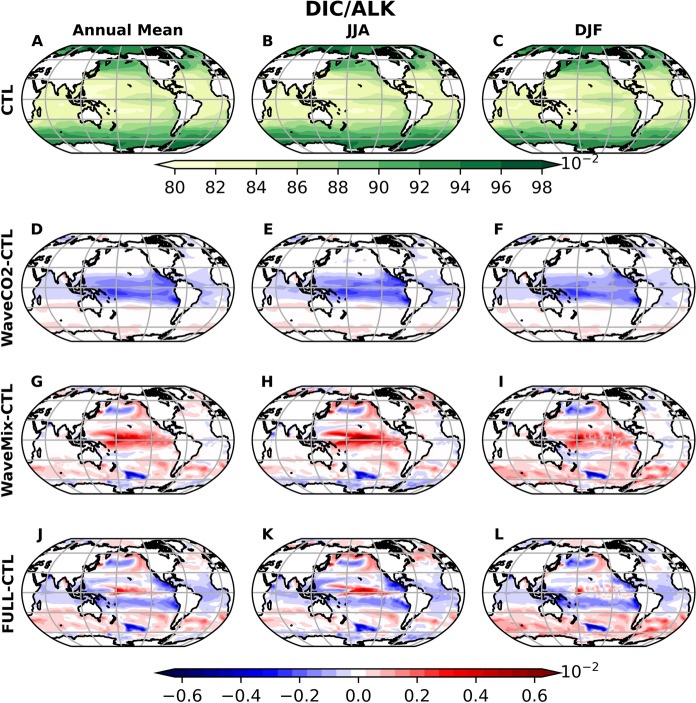
The global mean DIC/ALK from the CTL experiment (the first row) and the difference between the sensitivity experiments: WaveCO2-CTL (the second row), WaveMix-CTL (the third row), and FULL-CTL (the fourth row). The three columns represent the annual mean, JJA mean, and DJF mean. The blue in D-L represent the DIC/ALK is reduced, leading to the reduction of sea surface pCO$_2$, while red indicates the opposite

**Fig. 6:**
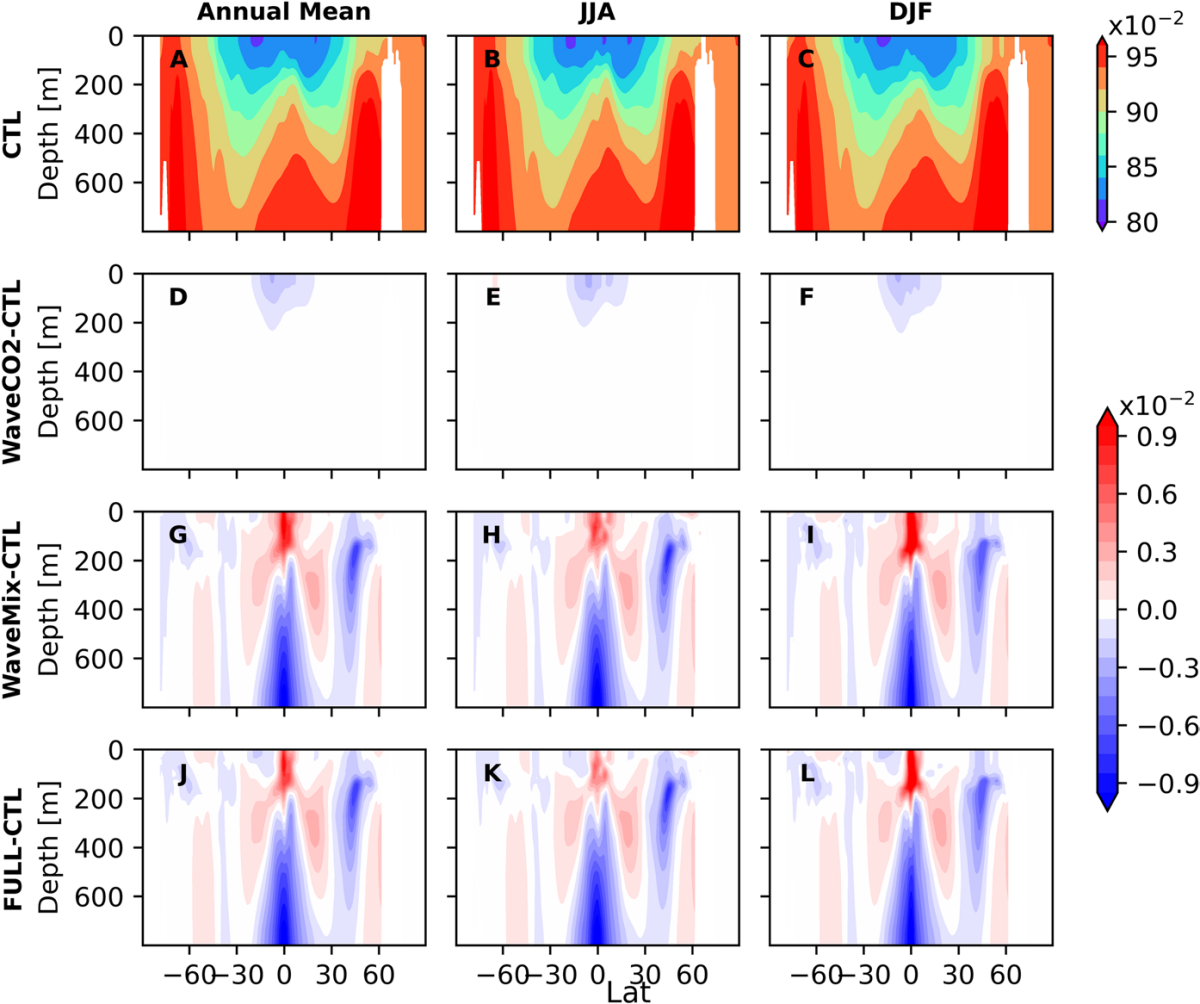
The cross section of the mean DIC/ALK from the CTL experiment (the first row) and the difference between the sensitivity experiments at 180$^o$ degree longitude: WaveCO2-CTL (the second row), WaveMix-CTL (the third row), and FULL-CTL (the fourth row). The three columns represent the annual mean, JJA mean, and DJF mean. The blue in D-L represent the DIC/ALK is reduced, while red indicates the opposite

The DIC/ALK ratio is higher in high latitudes (over 0.96) and lower in latitudes below 40$^{\circ }$. Additionally, there is a relatively high DIC/ALK ratio near the equator of the Pacific Ocean, contributing to areas with high sea surface pCO$_2$ (Fig. 5A-C). Compared to the CTL experiment, WaveCO$_2$ reduces the DIC/ALK ratio in the tropical region (the blue area in Fig. 5D–F), with this reduction extending vertically down to a depth of 200 m (see Fig. 6D–F for the cross-section at 180$^{\circ }$ longitude as an example). This reduction is resulted by the decrease of DIC concentration (warm color area in Fig. S3D-F) caused by the carbon outgassing (red area in Fig. 3A-C). Although the CO$_2$ flux difference between WaveCO2 and CTL experiments varies with the seasons, its decrease on the sea surface DIC/ALK ratio shows little seasonal variation. This is attributed to the advection of ocean tracers, including DIC and ALK, by major ocean currents such as the Equatorial Countercurrent, North Equatorial Current, and South Equatorial Current, which act to reduce the horizontal gradient of DIC.

Compared to WaveCO2 where the sea surface DIC in tropical areas is slightly reduced, the wave-current interaction processes (WaveMix) lead to a pronounced increase in both the sea surface DIC and ALK in the tropical areas of the Pacific Ocean (between 30$^\circ$N and 30$^\circ$S in Figs. S3 and S4). This relatively larger influence on DIC increases the DIC/ALK ratio (red area in Fig. 5G-I), positively affecting pCO$_2$ in these areas (red area in Fig. 4G-I). Unlike the WaveCO$_2$ flux, which primarily affects the DIC/ALK ratio in the upper 200 m, wave–current interaction processes can impact the ratio at depths greater than 1000 m and reduce its vertical gradient (Fig. 6), due to enhanced upper-ocean mixing. The enhancement of surface DIC/ALK ratio caused by wave-current interaction processes is stronger in June-July-August (JJA) than in December-January-February (DJF). In the Antarctic, there is a slight decrease in both surface DIC and ALK (area with warm color around the Antarctic in Figs. S3 and S4). The reduction of DIC is more pronounced in JJA than in DJF, leading to a decrease in the DIC/ALK ratio in JJA but an increase in DJF (Fig. 5). This indicates that changes in the DIC/ALK ratio caused by wave-current interaction processes favor reducing ocean surface pCO$_2$ during JJA and increasing pCO$_2$ in DJF around the Antarctic. In the central North Pacific Ocean, wave–current interaction processes lead to a pronounced increase in ALK, as indicated by the green area in Fig. S4G–I. This increase results in a reduction of the DIC/ALK ratio (blue area in Fig. 5G–I), which extends vertically to depths greater than 600 m (Fig. 6G–I). Consequently, in this area, there is a reduction in pCO$_2$, which enhances CO$_2$ uptake (Figs. 3 and 4). The variation of ALK in this area is primarily caused by altered mixing in the upper ocean due to ocean waves.

Although the influence of wave-dependent gas transfer on surface DIC is relatively small (WaveCO2–CTL; Fig. S3), the resulting changes in the DIC/ALK ratio are comparable to those induced by wave-current interaction processes (WaveMix–CTL) (Fig. 5D–I), as surface ALK remains unchanged in the WaveCO2 experiment. Therefore, the combined effect of the two different processes exhibits (FULL-CTL) pronounced spatial and seasonal variability. In the equatorial region, the difference between FULL and CTL is largely driven by wave-current interaction processes, enhancing the pCO$_2$. This is because the vertical gradient of the DIC/ALK ratio in the equatorial region is larger than in other regions (Fig. 6A–C). Consequently, the enhanced upper-ocean mixing induced by wave–current interaction processes leads to a more pronounced alteration of the DIC/ALK ratio. In contrast, in the subtropical regions (approximately 10$^{\circ }$–30$^{\circ }$ latitude), changes in the DIC/ALK ratio are mainly attributed to the sea-state-dependent gas transfer parameterization (Fig. 5), reducing the surface pCO$_2$. These findings highlight the importance of considering both mechanisms in coupled ocean biogeochemical models to better capture the spatial heterogeneity in air-sea CO$_2$ exchange.

### SST

SST affects the air-sea CO$_2$ flux by altering the CO$_2$ solubility. Lower SST increases CO$_2$ solubility, thereby decreasing the sea surface pCO$_2$. In contrast, higher SST will decrease CO$_2$ solubility and increase the pCO$_2$ in the surface water. Since there is no SST difference between CTL and WaveCO2 (or between FULL and WaveMix), we focus the following analysis on the difference between WaveMix and CTL.

**Fig. 7:**
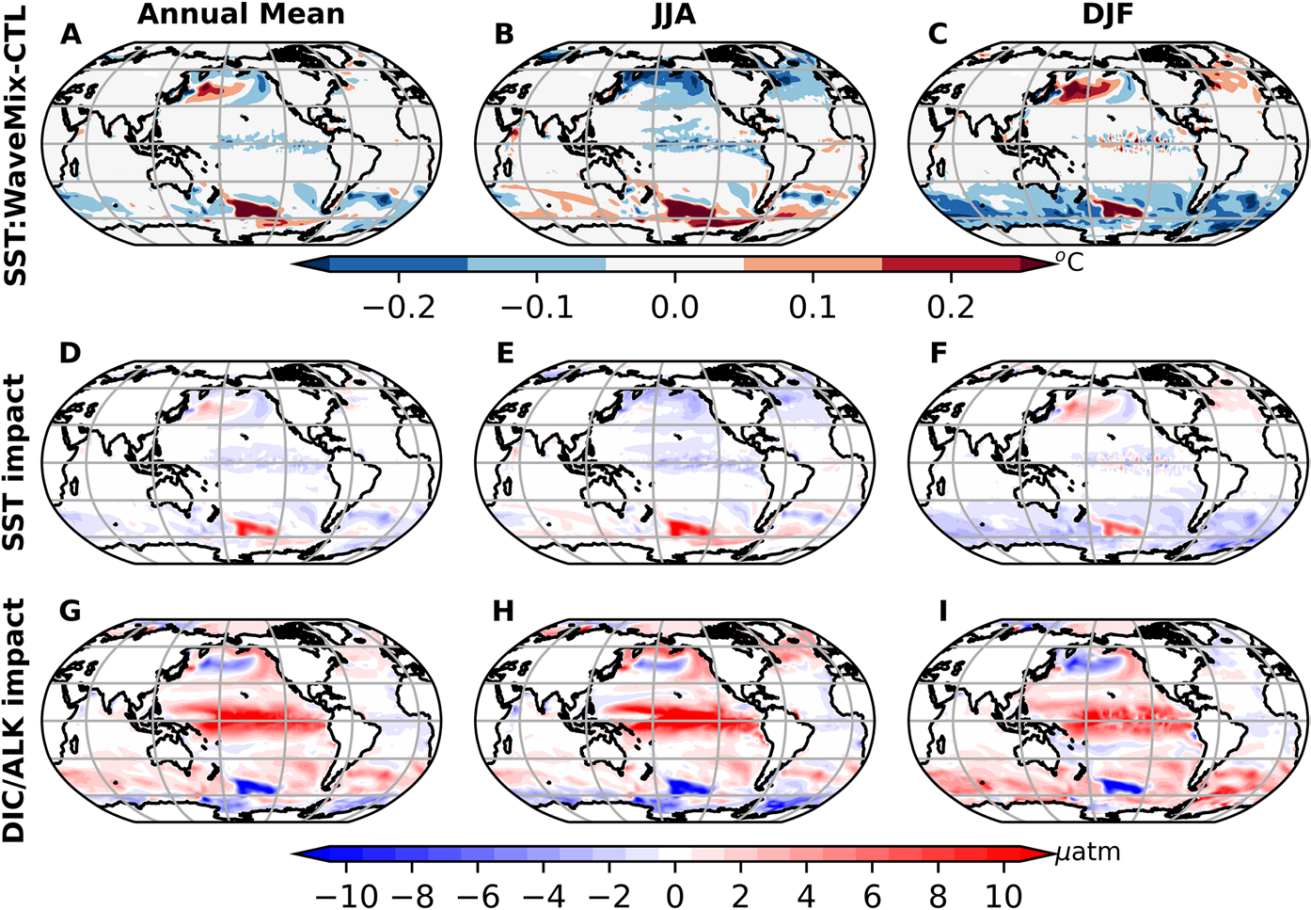
The first row shows the impact of wave–current interaction processes on SST (WaveMix–CTL) [unit: $^\circ$C]. The second and third rows illustrate the changes in $\Delta$pCO$_2$ resulting from variations in DIC/ALK and SST, respectively, with units in $\mu$atm. The three columns represent the annual mean, JJA, and DJF averages. In panels D–I, blue indicates the negative impact on the ocean surface pCO$_2$, while red indicates the opposite

Wave-current interaction processes generally decrease annual mean SST around the equatorial Pacific and regions higher than 30$^\circ$ latitude, except in the southeast areas of New Zealand and the northern Pacific with high wind speeds. The influence of wave-current interaction processes varies markedly by season. Except in the southeast areas of New Zealand, wave-current interaction processes (WaveMix) typically reduce SST in summer but increase it in winter (Fig. 7A-C). In winter, the ocean surface loses heat, facilitating the reduction of SST. The enhanced upper ocean mixing induced by wave-current interaction processes can compensate for the heat lost from the lower water, leading to a higher surface temperature. Conversely, in summer, the enhanced mixing by waves more effectively mixes the surface warm water within the mixed layer, reducing SST. The SST in the southeast areas of New Zealand is increased by upper-ocean mixing processes all year around. This is mainly because the altered ocean circulation happened in these areas by surface waves. Compared to the control experiment (CTL), WaveMix enhances the southeastward heat transport in the southeast areas of New Zealand all year around (Figures are not shown). Given the pronounced SST gradients, a small shift in ocean currents can lead to significant SST changes in this area.

### Sea ice

Sea ice extent affects the air-sea CO$_2$ flux by decreasing the open water areas, since there is no CO$_2$ flux over ice in the current model set-up. Meanwhile, the ice melting and freezing will also alter the distribution of DIC and ALK, thus, affecting the pCO$_2$ and CO$_2$ flux. The sea ice extent from WaveCO2 (FULL) is identical to that from CTL (WaveMix). Therefore, in the following analysis, we focus solely on the differences between WaveMix and CTL.

**Fig. 8:**
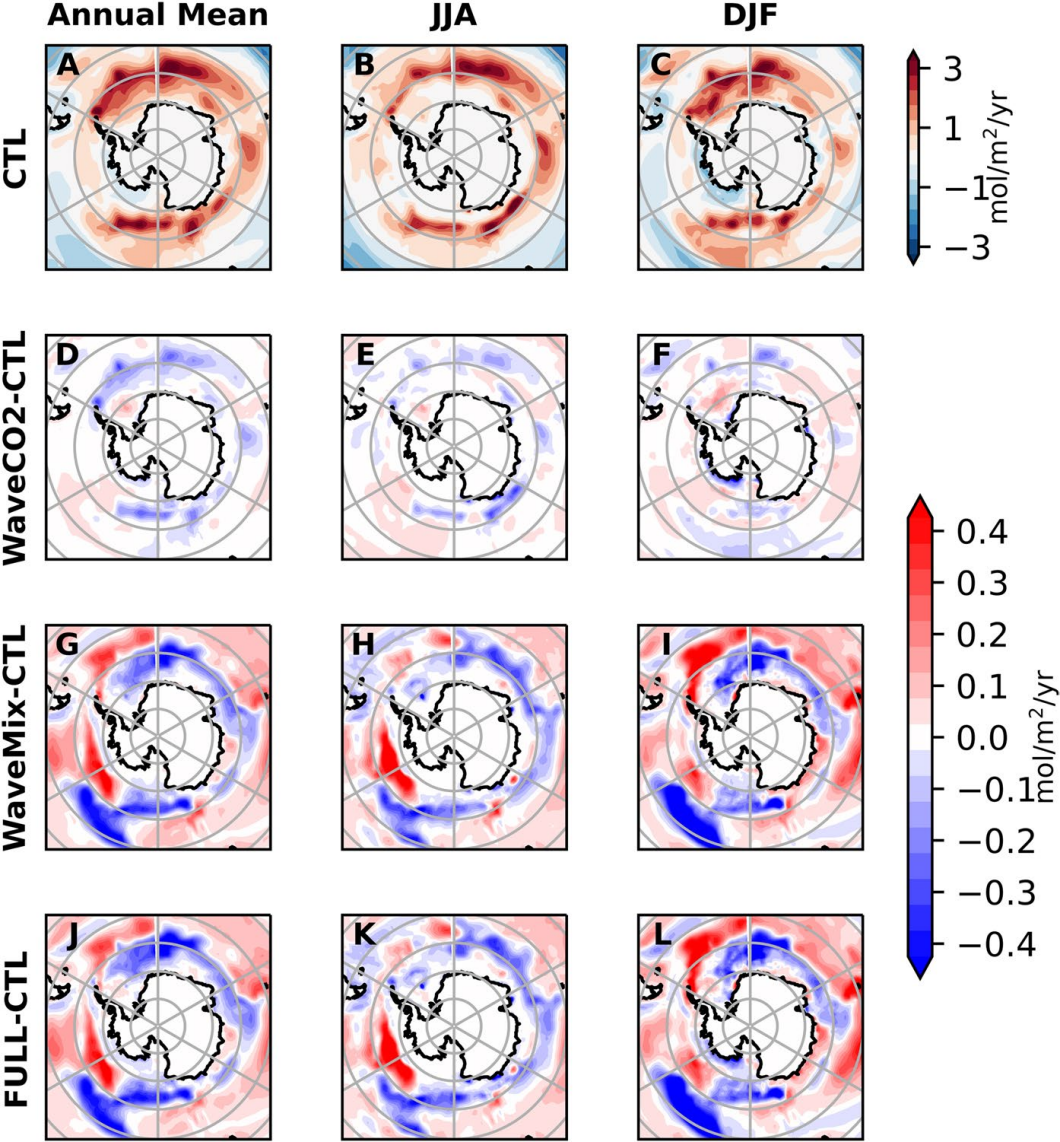
Mean CO$_2$ flux and experimental differences in the Antarctic: The first row shows the mean CO$_2$ flux from the CTL experiment. The second to fourth rows display the differences between the sensitivity experiments and CTL: WaveCO2–CTL, WaveMix–CTL, and FULL–CTL, respectively. The three columns represent the annual mean, JJA (June–July–August) mean, and DJF (December–January–February) mean [unit: mol/m$^2$/yr]. In panels A–C, red (blue) indicates carbon outgassing (uptake). In the other panels, red shading indicates either enhanced CO$_2$ outgassing flux or weakened CO$_2$ uptake flux, while blue indicates the opposite

The wave-current interaction processes have little impact on the sea ice in the Arctic. Thus, we will focus on their impact on Antarctic (Fig. 8). During the Antarctic winter, wave-current interaction processes reduce sea ice concentration by up to 10% in the Amundsen Sea. In contrast, they increase sea ice concentration by more than 10% during the Antarctic summer in the Weddell Sea (Fig. S5). This influence on sea ice concentration is directly linked to upper ocean temperature, which decreases during summer but increases during winter. Consequently, it contributes to sea ice reduction in winter and increases in summer.

In most areas of the Antarctic, the ocean acts as a carbon source to the atmosphere (Fig. 8). The primary difference between WaveMix and CTL is observed in the marginal ice zone (MIZ) area, where sea ice melts and freezes seasonally. During JJA, wave-current interaction processes expose more water surfaces (i.e., reducing sea ice concentration; Fig. S5), allowing carbon to transfer from the ocean to the atmosphere. Additionally, higher SST decreases CO$_2$ solubility and increases pCO$_2$ in the surface water. Conversely, the reduction of the ice-free area indicates that there will be fewer impurities rejected from the ice, leading to a lower DIC/ALK ratio compared to open seawater, which favours lower pCO$_2$ and thus reduces CO$_2$ flux to the atmosphere. At the same time, the enhanced mixing by wave-current interaction processes also reduces the DIC/ALK ratio in the Antarctic. Among these factors, the change in DIC/ALK is the dominant one. Consequently, these factors collectively reduce the carbon flux from the ocean to the atmosphere, with some small areas experiencing carbon flux from the atmosphere to the ocean (Fig. 8).

In DJF, wave-current interaction processes reduce the SST, which increases CO$_2$ solubility, thereby decreasing the pCO$_2$ in the ocean surface water. Meanwhile, the increase in sea ice concentration caused by lower SST reduces the water surface area, negatively influencing the carbon outgassing. The reduced sea-ice meltwater compared to the CTL experiment leads to a relatively higher DIC/ALK ratio in the local area, as sea-ice meltwater has a lower DIC/ALK ratio. This relatively lower DIC/ALK potentially reduces the pCO$_2$ locally, thus decreasing the air-sea CO$_2$ flux. Overall, these factors reduce the carbon outgassing in the Antarctic during DJF (Fig. 8).

## Discussion

The traditional wind-speed-dependent CO$_2$ gas transfer velocity parameterization is often found to underestimate the air-sea CO$_2$ flux (Zhou et al. 2023). By explicitly considering the bubble-mediated gas transfer velocity, the sea-state-dependent parameterization developed by Deike and Melville (2018) can better capture the air-sea CO$_2$ flux (Zhou et al. 2023). Unlike previous studies (Zhou et al. 2023; Sun et al. 2023) that estimate the air-sea gas transfer velocity or CO$_2$ flux offline, we investigate the influence within a coupled model to better capture the indirect effects of changes in surface DIC, driven by air-sea CO$_2$ flux, on the air-sea CO$_2$ flux itself. Our simulation results indicate that the sea-state-dependent gas transfer velocity parameterization, including bubble-mediated terms, strongly influences the global CO$_2$ flux, with variations across seasons and locations. The results are consistent with Rustogi et al. (2024), based on simulations for the period 1959–2018: wave-dependent gas transfer velocity enhances carbon outgassing in the equatorial region and reduces (increases) carbon outgassing (uptake) in regions extending up to 30$^{\circ }$ latitude. In our study, where the atmospheric CO$_2$ partial pressure is set to preindustrial conditions, the spatial patterns affected by sea-state-dependent gas transfer velocity show some differences. Additionally, we found that the direction of the impact in the 10$^{\circ }$–35$^{\circ }$ latitude range exhibits pronounced seasonal variation, with an enhancement of carbon uptake during winter.

Compared to the sea-state-dependent parameterization gas transfer velocity parameterization that explicitly accounts for bubble-mediated processes, wave–current interactions enhance upper ocean mixing, which in turn alters the DIC/ALK ratio and SST, subsequently affecting pCO$_2$ and the air–sea CO$_2$ flux. The effect of DIC/ALK changes on $\Delta$pCO$_2$ generally opposes that of SST changes (Fig. 7D–I). Furthermore, the magnitude of the SST contribution is typically smaller than that of DIC/ALK, except in regions such as the northern North Pacific and the southeastern area near New Zealand, where SST changes exceed 0.3$^\circ$C. In addition to the wave-current interaction processes tested in this study (Section 2.3), other wave-related processes may also play a potential role in air-sea gas exchange, such as nonbreaking wave-induced mixing (Qiao et al. 2004). Furthermore, these wave-current interaction processes are currently implemented in an offline coupled mode in this study, where the ocean/ice does not affect the wave. The next step involves using an online coupled wave-ocean-ice-biogeochemistry model to test the coupling influence.

The CO$_2$ flux over the Southern Ocean and the Antarctic is sensitive to the wave-current interaction processes. This is mainly because the wind speed and Stokes drift are higher in these areas and the redistribution role of waves on the wind stress is significant (Wu et al. 2022). Meanwhile, the influence of wave processes on the seasonal variation of the MIZ is another factor contributing to the changes in CO$_2$ flux in the Antarctic. It is important to note that in the NEMO model, the CO$_2$ flux in the MIZ is determined by the fraction of open water surface. Thus, the CO$_2$ flux changes linearly with the sea ice concentration. However, some studies indicate that the total CO$_2$ flux over a model grid changes non-linearly with the sea ice concentration (Loose et al. 2014). Meanwhile, there are gas fluxes between the air and the ice (Delille et al. 2014) which is not included in the current model.

With ongoing climate change, both wind speed and wave height are projected to increase (Young and Ribal 2019). These intensified conditions are expected to amplify the role of ocean surface waves in modulating the air-sea CO$_2$ flux. In particular, wave-driven processes–such as bubble-mediated gas transfer and indirect changes in pCO$_2$ caused by wave-current interactions–are likely to play a more important role compared to the relatively stable, preindustrial conditions examined in this study. Consequently, climate models that neglect or oversimplify these wave-related processes risk substantially underestimating or misrepresenting oceanic carbon uptake, leading to increased uncertainty in projections of the global carbon cycle and future climate trajectories. At the same time, the increasing resolution of climate and Earth system models now makes it both feasible and necessary to explicitly account for the influence of surface waves on air-sea CO$_2$ exchange. Advancing the integration of wave dynamics with biogeochemical components in climate models thus represents a critical step toward reducing uncertainties in long-term carbon budget assessments and enhancing predictions of climate-carbon feedback mechanisms.

In this study, preindustrial atmospheric CO$_2$ levels were used to isolate and better understand the physical mechanisms–particularly wave-related processes–that influence air–sea CO$_2$ exchange, without the added complexity of strong anthropogenic forcing. Under more realistic scenarios with increasing atmospheric CO$_2$ concentrations, both the magnitude and spatial patterns of air–sea CO$_2$ fluxes–as well as their sensitivity to wave processes–are likely to differ. For instance, in regions where CO$_2$ outgassing dominates, higher atmospheric CO$_2$ concentrations would reduce the $\Delta$pCO$_2$, thereby diminishing the influence of wave-related parameterizations compared to preindustrial conditions. Conversely, in areas characterized by net CO$_2$ uptake flux, rising atmospheric CO$_2$ levels would increase the magnitude of $\Delta$pCO$_2$, potentially amplifying the influence of wave-related processes. Incorporating time-varying CO$_2$ levels in future simulations will therefore be essential for assessing the interaction between wave processes and anthropogenic forcing, and for enhancing the predictive capability of Earth system models under evolving climate scenarios.

In the current study, the ORCA2 configuration of the NEMO model is used for sensitivity simulations. This coarse resolution simulation may miss some small-scale processes, leading to uncertainties in the results. For example, the sea ice coverage in these simulations may not accurately capture the finer details of ice dynamics and distribution. This limitation can affect the model’s ability to estimate precise air-sea carbon flux. Meanwhile, coarse resolution limits the model’s ability to accurately resolve ocean mixing and current structures, especially in regions with sharp gradients or mesoscale activity. This can lead to biases in the transport and distribution of key biogeochemical tracers such as DIC and ALK, which are essential for assessing air-sea CO$_2$ fluxes. Furthermore, in shelf and coastal regions–where wave breaking, coastal upwelling, and interactions between waves, currents, and bathymetry are more dynamic–the lack of spatial detail hinders the model’s ability to represent these processes realistically. This may result in an underestimation or misrepresentation of wave-induced vertical mixing and nearshore CO$_2$ exchange, both of which are important for regional carbon cycle assessments. Nevertheless, the simulation results provide significant insights into the influence of waves on the global air-sea carbon budget. Future studies with high-resolution and fully coupled Earth system models will be conducted.

## Conclusions

Ocean surface waves affect the air-sea CO$_2$ flux mainly in two ways: 1) by increasing the surface area available for gas exchange through air bubbles induced by wave breaking, and 2) by altering the sea surface pCO$_2$ and gas solubility through changes in upper ocean mixing (by wave-current interaction processes). The processes related to these influences are tested in an ocean-ice-biogeochemistry coupled model under preindustrial conditions by implementing wave-state-dependent parameterizations.

The simulation results indicate that both wave–current interaction processes and the sea-state-dependent gas transfer scheme–which explicitly accounts for bubble-mediated gas transfer velocity–strongly influence the air–sea CO$_2$ flux. The direction of their influence (i.e., whether they enhance or suppress gas transfer) and their relative contributions vary spatially and seasonally:

- In the equatorial region (10$^{\circ }$S–10$^{\circ }$N), both the sea-state-dependent gas transfer velocity parameterization and wave–current interaction processes enhance the CO$_2$ outgassing flux, with comparable magnitudes (more than 10% on average). The influence of surface waves on the air–sea CO$_2$ flux exhibits insignificant seasonal variation in this region.
- In the region between approximately 10 and 35 degree, the impact of ocean surface waves on the air-sea CO$_2$ flux through the sea-state-dependent gas transfer velocity is greater than that through the wave-current interaction processes, with opposite directions of influence. The effect of the sea-state-dependent gas transfer velocity shows significant seasonal variation, with the largest impact during the winter season. During winter, it enhances the carbon uptake flux, while in the summer season, it increases the carbon outgassing flux. In comparison, the seasonal variation of the wave-current interaction impact on air-sea CO$_2$ flux is less pronounced.
- In regions poleward of 35 degrees latitude, the influence of wave-current interaction processes is more significant than that of the sea-state-dependent gas transfer velocity, making the combined effect of both processes primarily driven by wave-current interactions. Additionally, the variation in sea ice concentration, caused by wave-current interaction processes, also contributes to the variation in CO$_2$ flux in the Antarctic region by affecting the open water area for air-sea gas exchange.

Although the CO$_2$ outgassing flux reduces surface pCO$_2$ (thereby suppressing the CO$_2$ outgassing flux), the enhanced CO$_2$ outgassing flux is primarily driven by the increase in air-sea gas transfer velocity. For the wave-current interaction processes, their impact on air-sea CO$_2$ flux through changes in pCO$_2$ is largely determined by the variations in DIC/ALK. In comparison, the changes in SST exert an opposite influence on pCO$_2$ relative to the DIC/ALK variation, but with a much smaller magnitude. From the sensitivity simulations under the preindustrial CO$_2$ condition, we can conclude that the wave-related processes impact on the air-sea gas exchange should be fully considered in Earth system models to better model the carbon cycle.

## Supplementary information

Below is the link to the electronic supplementary material.

Supplementary file 1 (docx 1688 KB)

## Data Availability

The NEMO code is accessed from https://www.nemo-ocean.eu/. The NEMO input data is from https://gws-access.jasmin.ac.uk/public/nemo/sette_inputs/index_RC.html.
