# Supplementary material for: Ocean surface waves impact on global air-sea CO2 flux
Source: Biogeochemistry. 2025 Sep 1;168(5):68. doi: 10.1007/s10533-025-01267-y (PMC12402038; doi:10.1007/s10533-025-01267-y)
Supplement: Supplementary file 1 — (docx 1688 KB) [file 10533_2025_1267_MOESM1_ESM.docx]

**Supporting information for “Ocean Surface Waves Impact on Global Air-Sea CO2 Flux”**

Lichuan Wu^1^, Yongqing Cai^2^ and Anna Rutgersson^1^

^1^Department of Earth Sciences, Uppsala University, Uppsala, 75236, Sweden.

^2^Laboratory for Polar Science of the Ministry of Natural Resources, Polar Research Institute of China, Shanghai, 200129, China.


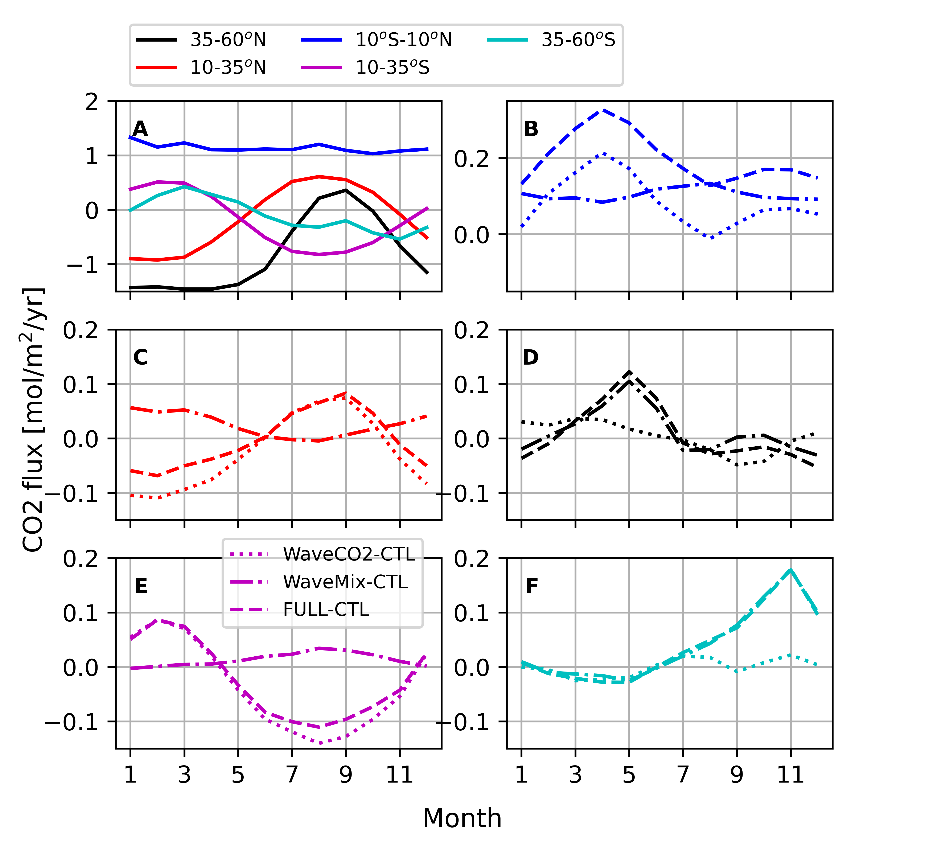


Fig. S1 Seasonal variation of the mean CO₂ flux across zonal bands: (A) shows the seasonal cycle, while (B–F) display the differences between sensitivity experiments and the control (CTL) experiment for the following regions: (B) 10°S–10°N, (C) 10°–35°N, (D) 35°–60°N, (E) 10°–35°S, and (F) 35°–60°S. Colors indicate different zonal bands, and line styles represent the differences between experiments.


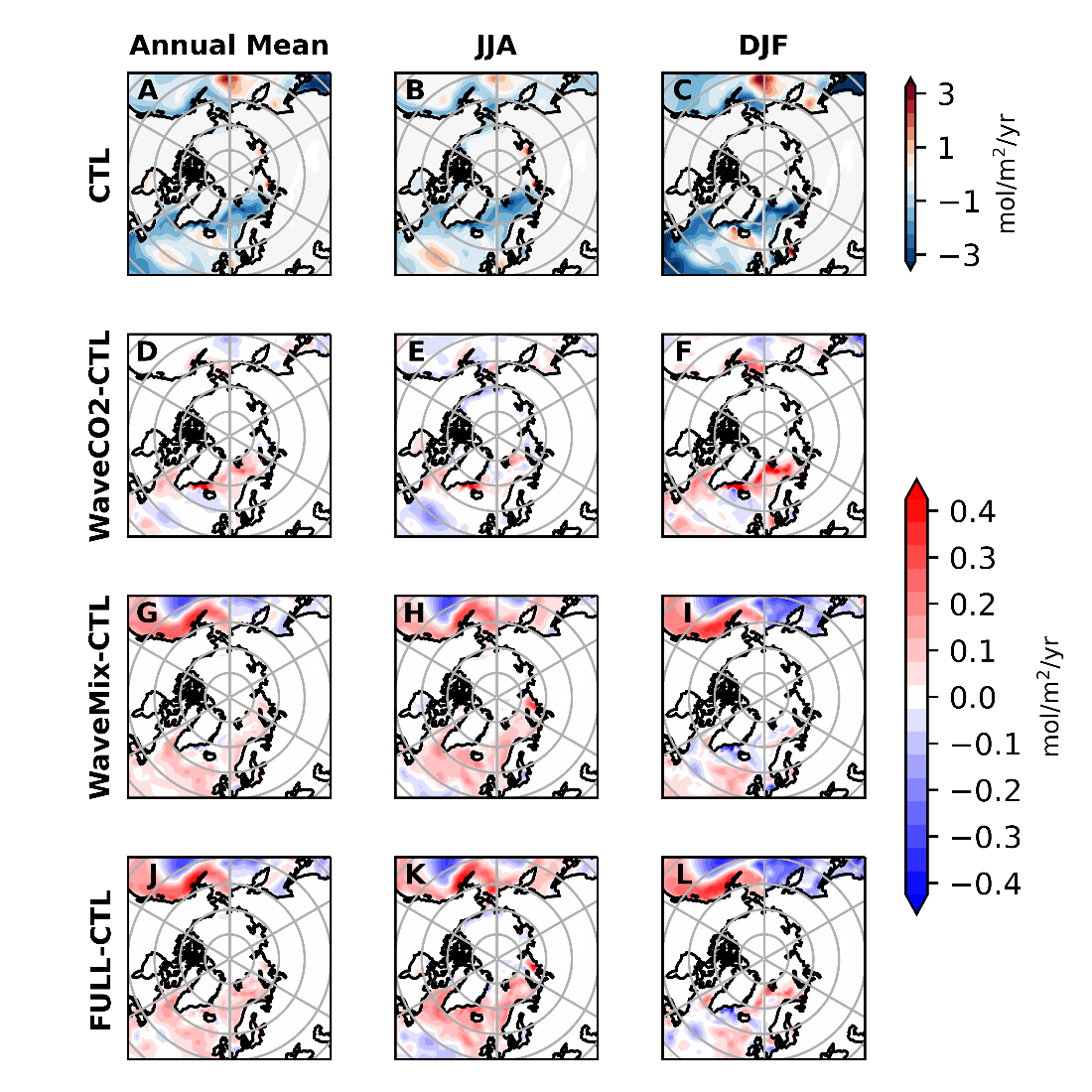


Fig. S2 Mean CO₂ flux and experimental differences in the Arctic: The first row shows the mean CO₂ flux from the CTL experiment. The second to fourth rows display the differences between the sensitivity experiments and CTL: WaveCO2–CTL, WaveMix–CTL, and FULL–CTL, respectively. The three columns represent the annual mean, JJA (June–July–August) mean, and DJF (December–January–February) mean [unit: mol/m²/yr]. In panels A–C, blue (red) indicates CO₂ uptake (outgassing). In the difference panels, red shading indicates either enhanced CO₂ outgassing flux or weakened CO₂ uptake flux, while blue indicates the opposite.


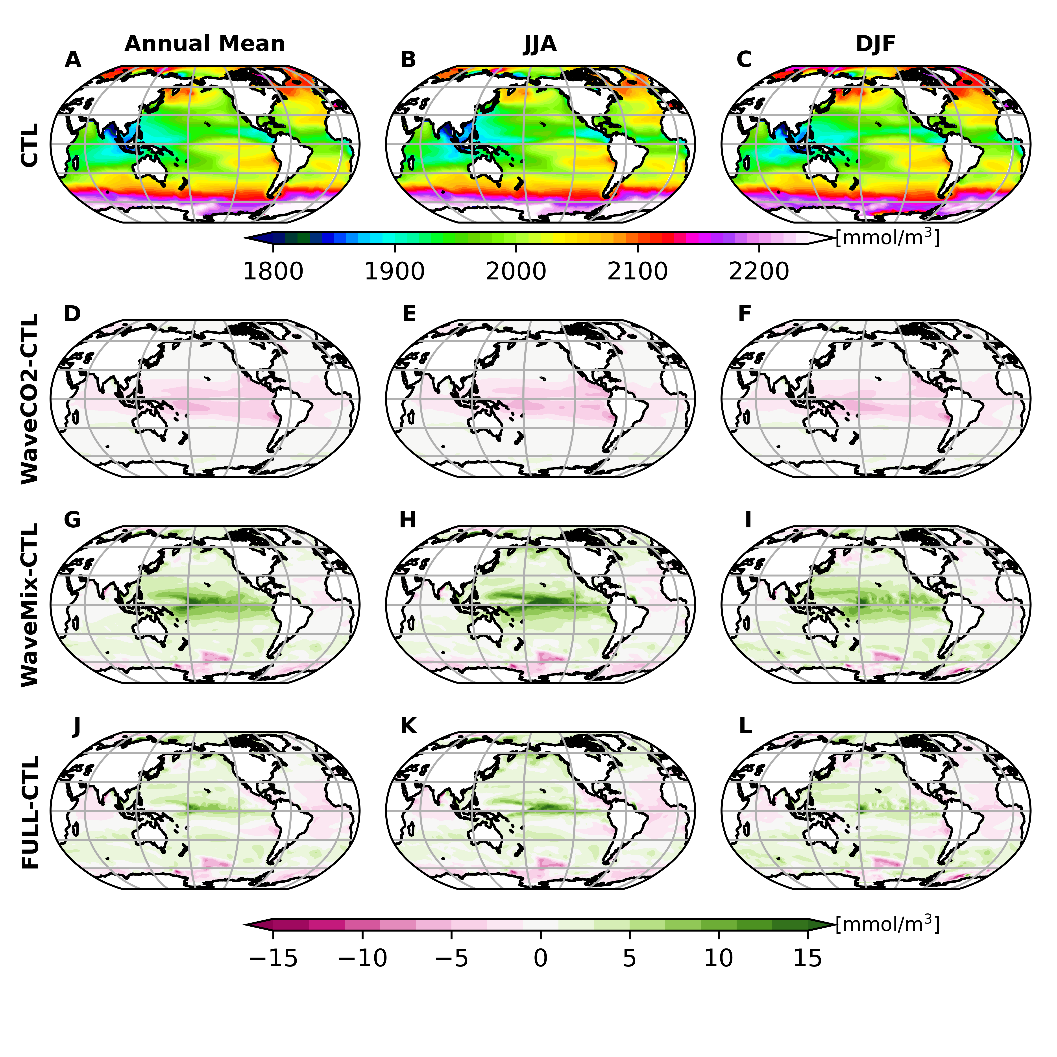


Fig. S3 The first row shows the global mean DIC from the CTL experiment, while the second to fourth rows display the differences between the sensitivity experiments and CTL: WaveCO2–CTL, WaveMix–CTL, and FULL–CTL, respectively. The three columns represent the annual mean, JJA mean, and DJF mean. Unit: [mmol/m³]


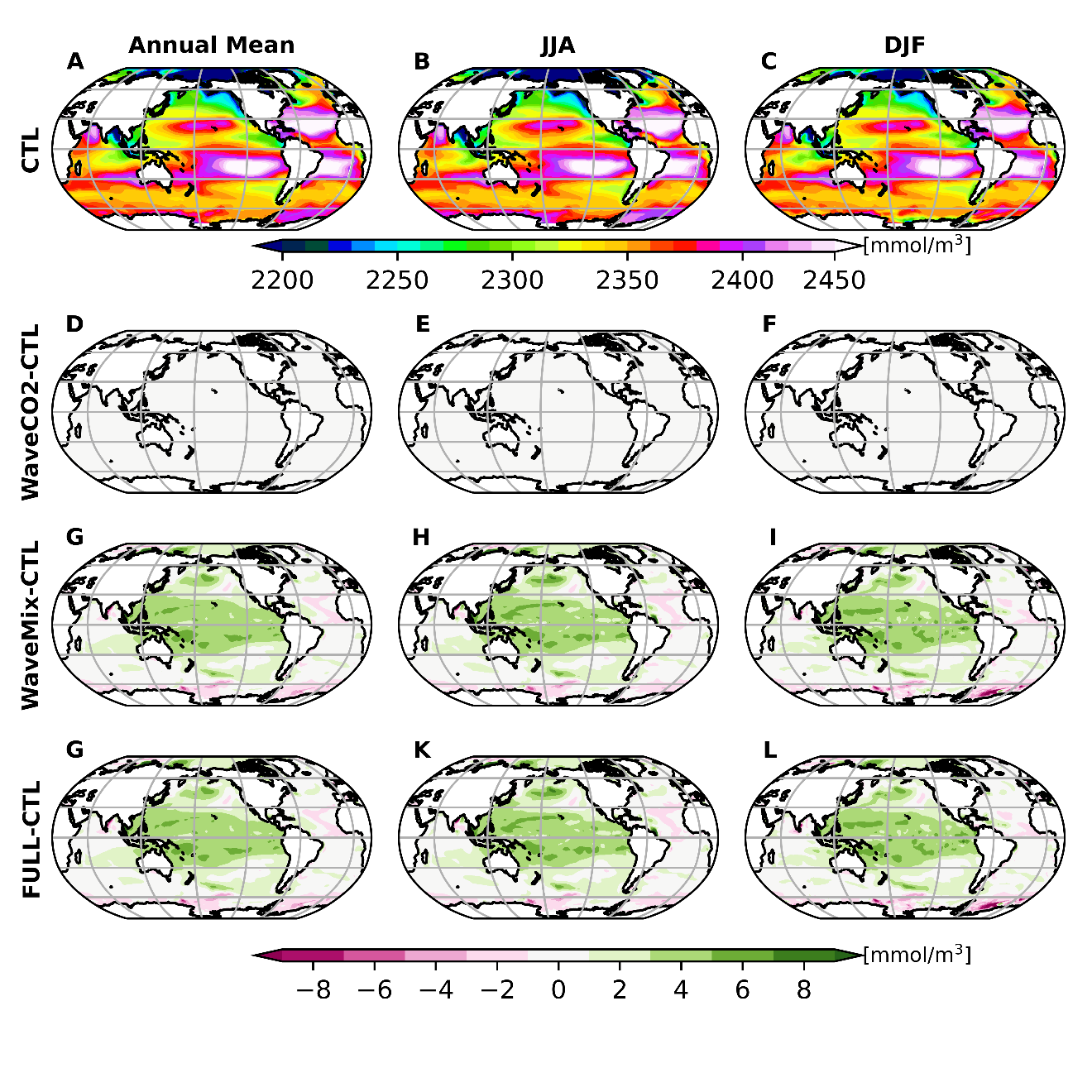


Fig. S4 The first row shows the global mean ALK from the CTL experiment, while the second to fourth rows display the differences between the sensitivity experiments and CTL: WaveCO2–CTL, WaveMix–CTL, and FULL–CTL, respectively. The three columns represent the annual mean, JJA mean, and DJF mean. Units: [mmol/m³]


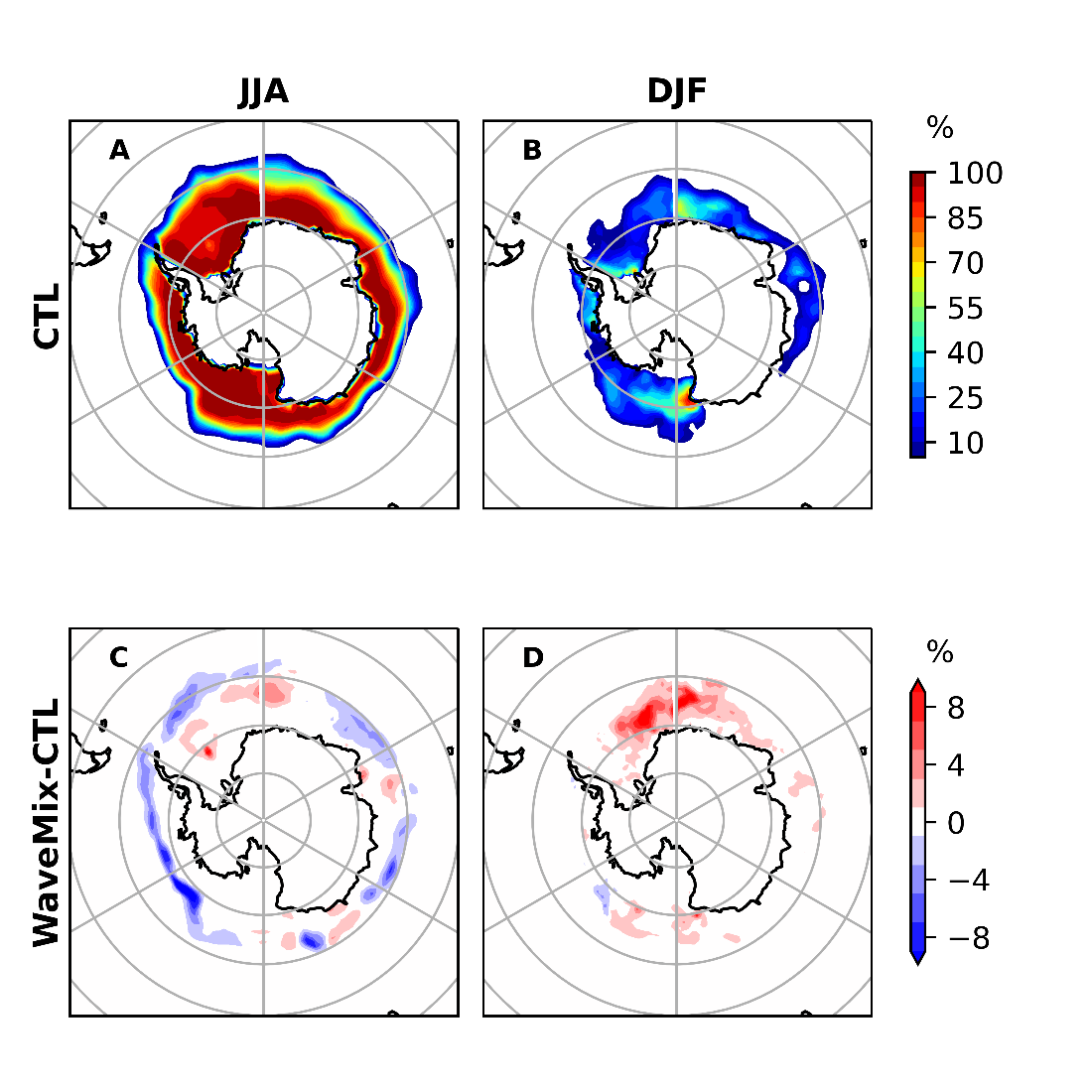


Fig. S5 The ice cover fraction in the Antarctic in JJA (A) and DJF (B) from the CTL experiment as well as the difference between WaveMix and CTL in JJA (C) and DJF (D) [unit: %].
